# A novel frequency-dependent lattice Boltzmann model with a single force term for electromagnetic wave propagation in dispersive media

**DOI:** 10.1038/s41598-023-38175-w

**Published:** 2023-07-10

**Authors:** Huifang Ma, Mingming Tang, Hao Ren, Wenyue Guo, Kexin Zhang, Yefei Chen, Wanshun Jiang, Ying Wang, Bin Wu

**Affiliations:** 1 College of Electronic and Information Engineering, Shandong University of Science and Technology, Qingdao, China; 2 School of Materials Science and Engineering, China University of Petroleum (East China), Qingdao, China; 3 School of Geosciences, China University of Petroleum (East China), Qingdao, China; 4 Research Institute of Petroleum Exploration and Development, Beijing, China; 5 The 41st Institute of China Electronics Technology Group Corporation, Qingdao, China

**Keywords:** Optical materials and structures, Optical physics, Optical techniques

## Abstract

Electromagnetic wave simulation is of pivotal importance in the design and implementation of photonic nano-structures. In this study, we developed a lattice Boltzmann model with a single extended force term (LBM-SEF) to simulate the propagation of electromagnetic waves in dispersive media. By reconstructing the solution of the macroscopic Maxwell equations using the lattice Boltzmann equation, the final form only involves an equilibrium term and a non-equilibrium force term. The two terms are evaluated using the macroscopic electromagnetic variables and the dispersive effect, respectively. The LBM-SEF scheme is capable of directly tracking the evolution of macroscopic electromagnetic variables, leading to lower virtual memory requirement and facilitating the implementation of physical boundary conditions. The mathematical consistency of the LBM-SEF with the Maxwell equations was validated by using the Champman-Enskog expansion; while three practical models were used to benchmark the numerical accuracy, stability, and flexibility of the proposed method.

## Introduction

The simulation of electromagnetic (EM) fields inside materials has always been an important topic in electrodynamics^1,2^, materials science and engineering. Specifically, in the development of organic nanomaterials and photonic crystals^3–5^, there is a strong motivation to provide accurate description of the EM responses of dispersive films for efficient photonic devices^6,7^. In the past decades, several schemes have emerged to attack the EM propagation problem^8–10^. For instance, the finite difference time domain (FDTD) method, first proposed by Yee^11^, is one of the most effective approaches^9^. For linear problems in homogeneous and isotropic media, the FDTD solution satisfies the computational demands as long as the medium used is non-dispersive. However, for frequency-dependent EM simulations in dispersive media, the FDTD does not meet the requirements, where the relative permittivity is explicitly dependent on frequency^12,13^. With the aim of appropriately accounting for the dispersive effect, alternative techniques have been introduced by the community, including the recursive convolution^13,14^ and the auxiliary differential equation method^15,16^. In addition to all the above method, the (FD)^2^TD method is a promising methods in dispersive media by combing the recursive convolution method and FDTD method^17,18^.

In recent decades, the lattice Boltzmann methods (LBM) have achieved great successes in the numerical investigation of diffusion^19,20^, flow^21^, waves^22^, and even quantum mechanics^23–25^. Mehrizi and Mohamad^25^ investigated the heat transfer in elliptical-triangular annuli using the lattice Boltzmann method; Afrouzi et al.^26^ proved that lattice Boltzmann method could be used to simulate the behaviors of particle transport in concentric annuli. Gao and Chen^27^ further confirmed the reliability of lattice Boltzmann method when simulate the melting process. LBM methods have been widely used to describe the EM propagation in non-dispersive media with either scalar^28^ or vectorial distribution functions^29,30^. Yet only a few attempts have been made to solve the EM equations in dispersive media using the LBM. Chen et al.^12^ used the pseudo permittivity and two force terms to simulate EM wave propagation in one-dimensional dispersive media. However, the accuracy of this model is strongly correlated to the selection of time step, while the evaluation of the force terms requires a two-step iteration.

In this work, we presented a new expression of Maxwell’s equation in isotropic dispersive medium and implemented a novel frequency-dependent lattice Boltzmann model with a single extended force term (LBM-SEF). Specifically, the permittivity does not rely on the time step in this case. Using the proposed method, we determine that the pseudo-permittivity is not required and the calculation becomes more efficient with the aid of a force term. Furthermore, the computational accuracy is improved when compared with that of the previous two-force term method under a special case in this study. Hence, this endows the proposed method with a significant advantage over the previous LBM methods for simulation problems with large grid simulations. Compared with FDTD, this novel LBM-SEF scheme does not require parameters from adjacent cells to calculate the equilibrium distribution and pseudo-equilibrium potential, which significantly reduced the difficulties for parallellization^22,24,28^. Apart from the 1D EM wave propagation problems assessed in this work, it is feasible to extend this new numerical scheme to 2D and 3D dispersive materials, similar to the Chapman–Enskog expansion procedure demonstrated in this work.

This paper is arranged as follows: In Sect. “Results”, two models are used to validate the newly proposed scheme by comparing it to the analytical and existing numerical solutions. Then, we examined electromagnetic wave propagation through a dispersive medium film. The effects of the film thickness of the dispersive medium, the frequency on the EM propagation and the EM propagation characteristics in the periodic medium are investigated in Sect. “Effects of film thickness and frequency on Gaussian modulated sinusoidal EM pulse”. And in section “Materials and methods**”**, detailed derivation is performed to prove the mathematical consistency between the proposed LBM-SEF method and Maxwell equations via the Chapman–Enskog expansion technique.

## Results

To validate the proposed LBM-SEF model, we have carried out three typical cases which have either analytical or numerical exact solutions. The simulation results indicate that the new model is capable of accurately reproducing the EM wave propagation in both dispersive and non-dispersive media. Subsequently, we proceed to create more intricate simulations.

### EM pulses in a non-dispersive media

**Figure 1:**
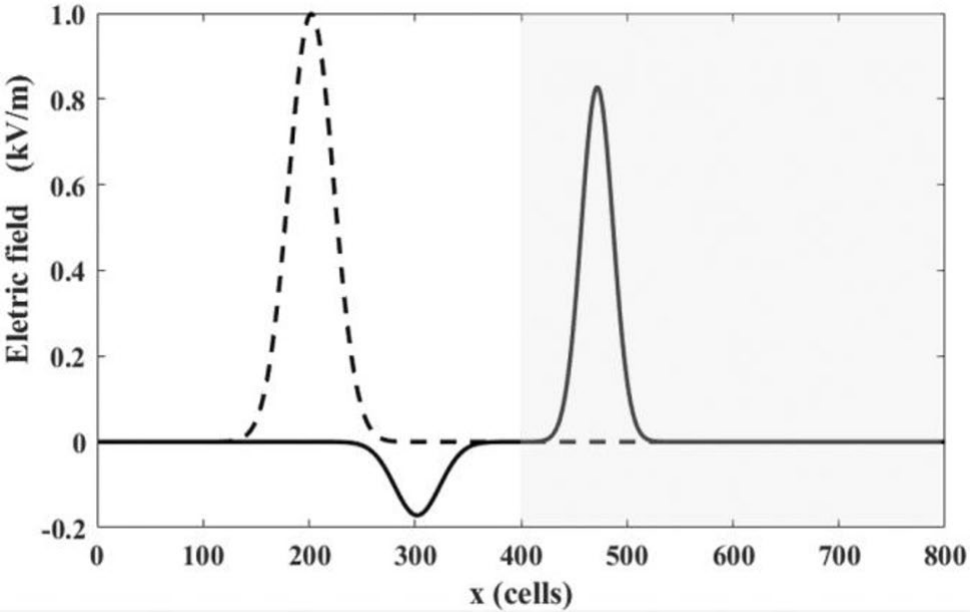
Distribution of electric pulse crossing a dielectric interface. The shadow zone is the dielectric medium, with relative permittivity constant of ${\varepsilon }_{r}=2.0$ and the other zone corresponds to the medium with ${\varepsilon }_{r}=1.0$. The curves correspond to the intensity of the electric field at t = 0 (dashed line) and t = 300 (solid line).

To conduct the initial benchmark, we conduct a simulation of a Gaussian pulse traveling through a one-dimensional array of $\mathrm{L}$ cells with periodic boundary conditions and crossing a dielectric interface. The first half of the simulation space, $x<\mathrm{L}/2$, represents a vacuum with a permittivity of ${\varepsilon }_{0}$, while the second half, $x>\mathrm{L}/2$, represents a non-dispersive medium with a relative permittivity constant of ${\varepsilon }_{r}=2.0$. The initial pulse is characterized according to previous studies^12,30^:

1 $$\mathrm{E}\left(x,t\right)={E}_{M}\mathrm{exp}(-{[(x-{x}_{c})/\alpha ]}^{2}), \mathrm{H}\left(\mathrm{x},\mathrm{t}\right)={H}_{M}\mathrm{exp}(-{[(x-{x}_{c})/\alpha ]}^{2})$$

where $\alpha$ is the pulse width, ${E}_{M}$ and ${H}_{M}$ are the amplitudes of electric and magnetic field of the pulse, respectively. We choose $\mathrm{L}=800$, ${E}_{M}=1000$, $\alpha =$ 30, and ${x}_{c}=250$. And continues boundaries are applied at the boundaries. The initial condition and the electric field at t = 300 are shown in Fig. 1.

The transmitted ${E}_{M}{\prime}$ and reflected pulses ${E}_{M}^{{\prime}{\prime}}$ can be evaluated using the incident pulse ${E}_{M}^{0}$^30,31^:

2 $$\frac{{E}_{M}{\prime}}{{E}_{M}^{0}}=\frac{2}{\sqrt{\frac{{\varepsilon }_{r}^{\mathrm{^{\prime}}}}{{\varepsilon }_{r}^{0}}}+1}, \frac{{E}_{M}^{^{\prime\prime} }}{{E}_{M}^{0}}=\frac{\sqrt{\frac{{\varepsilon }_{r}{\prime}}{{\varepsilon }_{r}^{0}}}-1}{\sqrt{\frac{{\varepsilon }_{r}^{\mathrm{^{\prime}}}}{{\varepsilon }_{r}^{0}}}+1}.$$

Based on Eq. (1), the theoretical amplitude ratio of the transmitted pulse and the incident pulse should be $\frac{{E}_{M}{\prime}}{{E}_{M}^{0}}=0.82843$, whereas that for the reflected pulse and the incident pulse should be $\frac{{E}_{M}^{{\prime}{\prime}}}{{E}_{M}^{0}}=0.17157$. Based on the proposed model, the computed values for these two ratios are 0.82761 and 0.17153, respectively. Hence, the computed values are in good agreement with the theoretical ones and the relative errors are less than 1‰.

**Figure 2:**
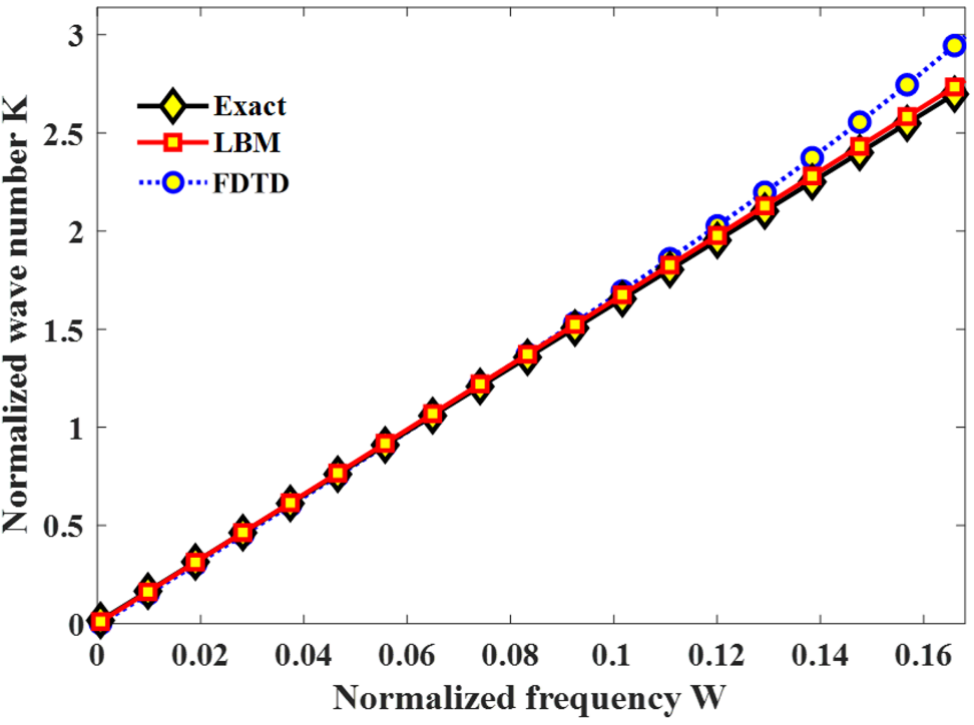
The dispersion relations generated by the FDTD [39] and the new LBM method compared with exact analytical solutions in non-dispersive media.

Based on the work of Dhuri et al.^32^, we delved deeper into the dispersion properties of the 1D LBM-SEF model by examining the response to plane waves in homogeneous, non-dispersive media. Figure 2 compares the dispersion relations generated by our model with those of previous FDTD^33^ simulations. The normalized wave number $K=2k\Delta x$, is displayed as a function of the normalized frequency,$W=\mathrm{\omega \Delta }t$, in Fig. 2.

As shown in Fig. 2, the dispersion relation of our LBM method is in good consistency with the exact analytical curve. Additionally, Fig. 2 suggests that the new LBM method has lower dispersion error than the FDTD method for high wave numbers ($$K>2.0)$$, which is further advantage of the LBM over FDTD method for the specific problem being discussed. On the other hand, the 1D LBM method uses three distribution functions ($${f}_{0}\left(x,t\right)$$, $${f}_{1}\left(x,t\right)$$,$${f}_{2}\left(x,t\right)$$) to represent two physical fields (**H** and **E**), thus requiring ~ 33% more memory compared to the 1D FDTD method.

### EM wave in Debye medium based on LBM method

In this case, we considered an electrical pulse propagating from air into water. In this case, water is treated as a Debye medium. For water, the properties for the relative permittivity are well described by the Debye model^31,34^ with ${\varepsilon }_{s}=81$, ${\varepsilon }_{\infty }=1.8$, and ${t}_{0}=9.4\times {10}^{-12}$ (Fig. 2). It is worth to be noted that, from the Debye model^31,34^

3 $$\upvarepsilon \left(\upomega \right)={\varepsilon }_{\infty }+\frac{{\varepsilon }_{s}-{\varepsilon }_{\infty }}{1+j\omega {t}_{0}},$$

where $\upvarepsilon \left(\upomega \right)$ denotes the relative permittivity as a function of frequency, we can calculate the real part $\mathrm{R}\left[\upvarepsilon \left(\upomega \right)\right]$ and imagery part $\mathrm{Im}\left[\upvarepsilon \left(\upomega \right)\right]$ of the relative permittivity as:

4 $$\mathrm{R}\left[\upvarepsilon \left(\upomega \right)\right]={\varepsilon }_{\infty }+\frac{{\varepsilon }_{s}-{\varepsilon }_{\infty }}{1+{\omega }^{2}{t}_{0}^{2}},\mathrm{ Im}\left[\upvarepsilon \left(\upomega \right)\right]=({\varepsilon }_{s}-{\varepsilon }_{\infty })\frac{\omega }{1+{\omega }^{2}{t}_{0}^{2}}$$

**Figure 3:**
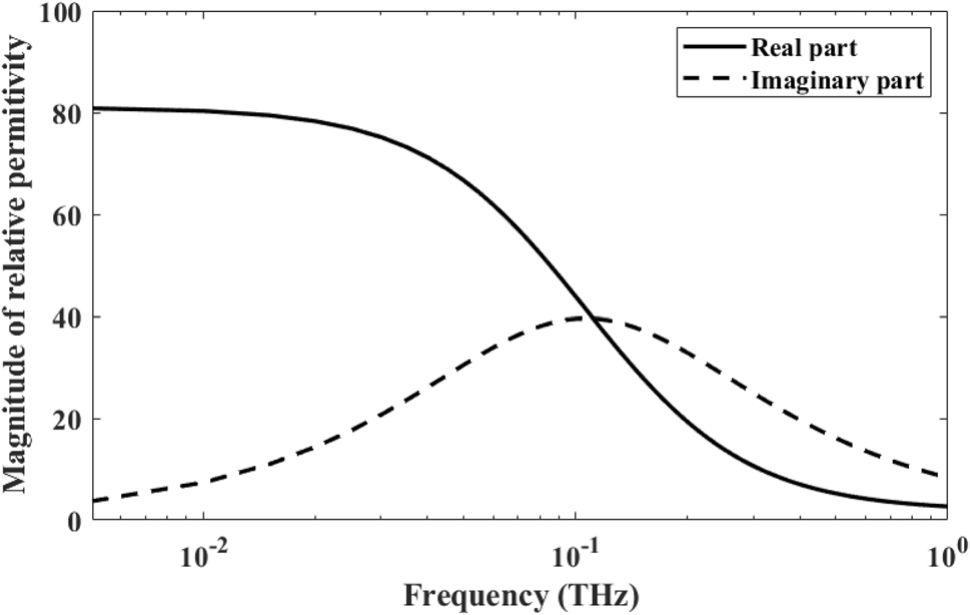
Complex relative permittivity of water in frequency domain.

Equation (4) shows that $\mathrm{R}\left[\upvarepsilon \left(\upomega \right)\right]$ decreases as the frequency of EM wave increases from low frequency to high frequency, whereas $\mathrm{Im}\left[\upvarepsilon \left(\upomega \right)\right]$ exhibits a bell shape while the frequency changes from low frequency to high frequency. Furthermore, the peak value of $\mathrm{Im}\left[\upvarepsilon \left(\upomega \right)\right]$ could be calculated as follows:

5 $$\frac{d\mathrm{Im}\left[\upvarepsilon \left(\upomega \right)\right]}{d\upomega }=\left({\varepsilon }_{s}-{\varepsilon }_{\infty }\right)\frac{1-{\omega }^{2}{t}_{0}^{2}}{{\left(1+{\omega }^{2}{t}_{0}^{2}\right)}^{2}}=0,{\upomega }_{max\upvarepsilon }=\frac{1}{{t}_{0}},$$

where ${\upomega }_{max\upvarepsilon }$ is the frequency that gives the maximum $\mathrm{Im}\left[\upvarepsilon \left(\upomega \right)\right]$. For water, as shown in Fig. 3, ${t}_{0}=9.4\times {10}^{-12}$, which gives ${\upomega }_{max\upvarepsilon }$=0.1063 THz.

**Figure 4:**
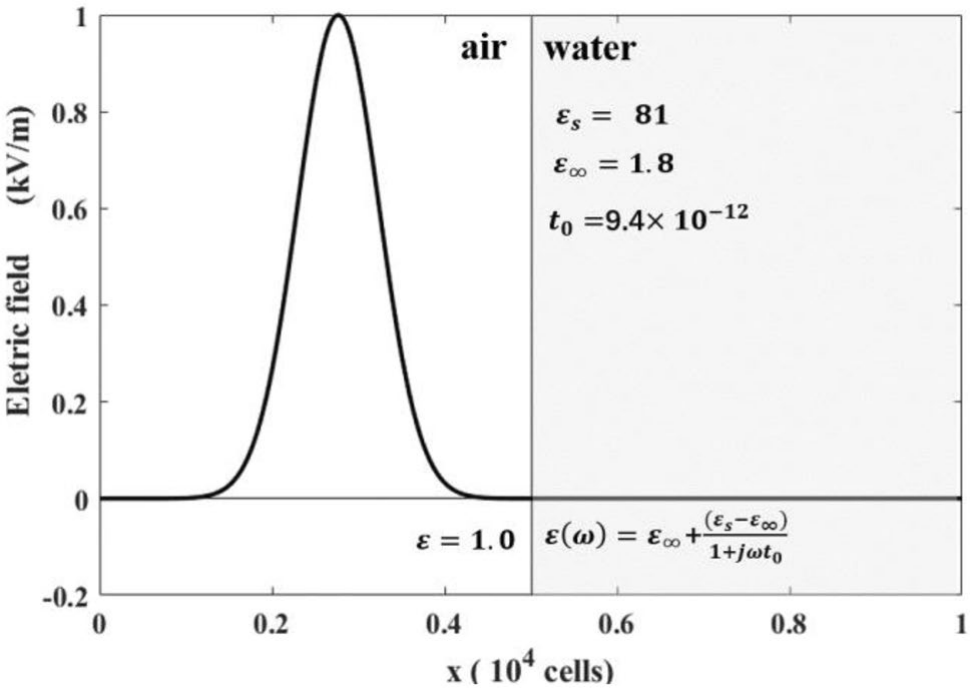
Plot of the electric field of the incident Gaussian pulse versus cell number at the beginning of the computations.

**Figure 5:**
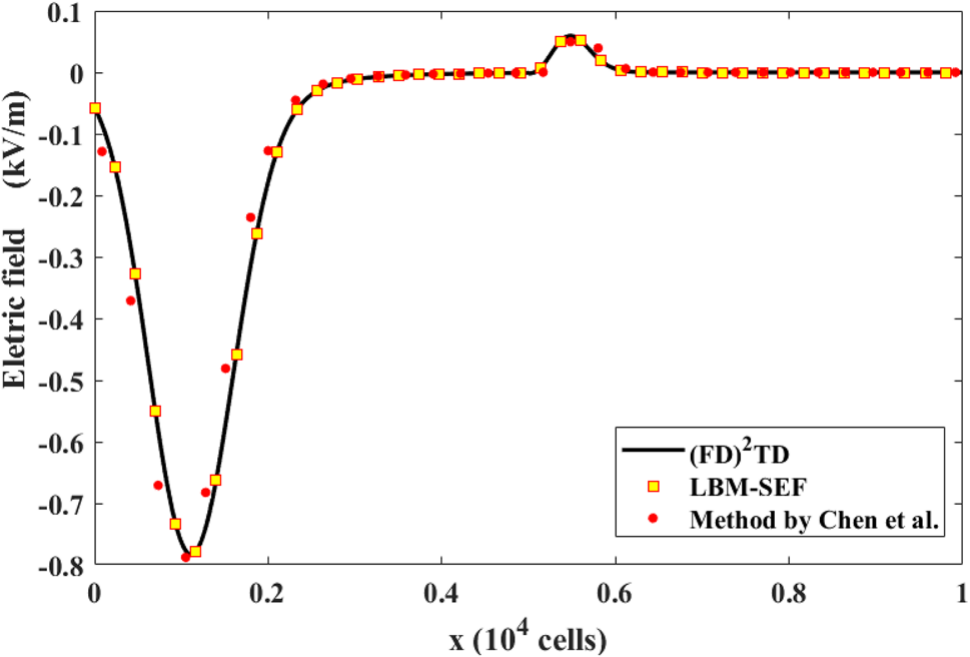
Comparisons of the numerical results obtained via the LBM-SEF method, (FD)^2^TD method^18^**,** and method proposed by Chen et al.^12^ at time = 75 ps.

Figure 4 depicts the initial electric field in the air. The computational domain consists of 800 lattices, with the left 400 for free space and the right 400 occupied by water. We used an incident pulse with a maximum amplitude of 1 kV/m and a width of 30 cells. The lattice size was set to 3.75 $\mathrm{\mu m}$, and the time step was 0.125 ps. These parameters have been used by Luebbers et al.^18^ and Chen et al.^12^ In Fig. 5, we compared our numerical results with previous studies generated by the well-established (FD)^2^TD and the LBM with pseudo permittivity schemes. It is notable that our results are more consistent with the accurate (FD)^2^TD results than the pseudo permittivity approach.

When dealing with conservative differential equations, it is important to give an estimate of the order of convergence of the model. This can be realized by monitoring the accuracy of the physical variables, specifically, the EM energy density, and the resolution of the lattice grid is increased while keeping the time resolution constant:

6 $$\mathrm{U}=\frac{1}{2}\left({\varepsilon }_{r}\overrightarrow{E}.\overrightarrow{E}+\frac{1}{{\mu }_{R}}\overrightarrow{B}.\overrightarrow{B}\right).$$

**Figure 6:**
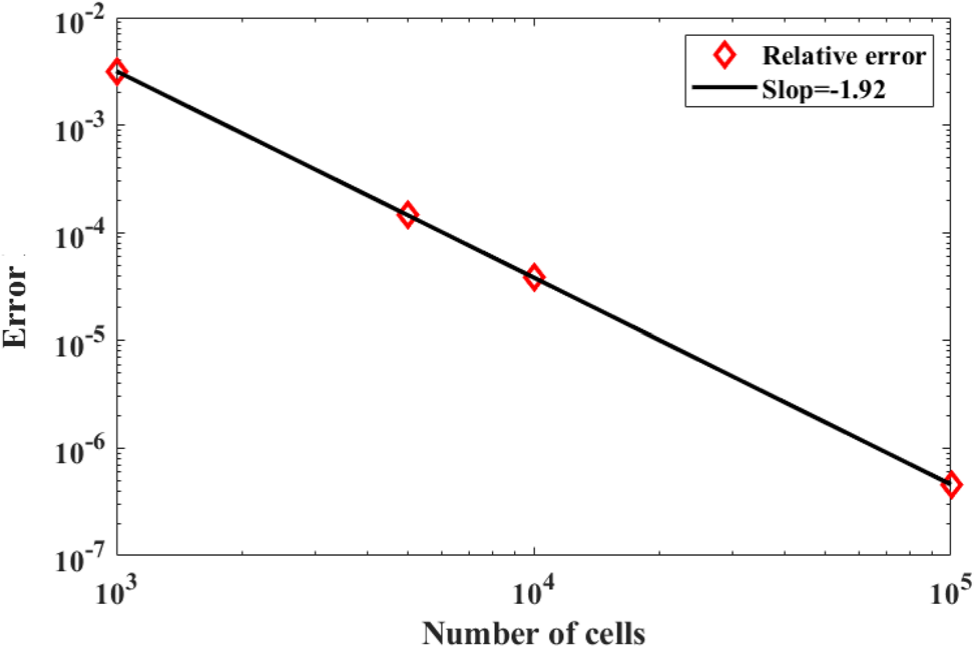
Numerical error as a function of the number of grid points.

Figure 6 depicts the EM energy density U as a function of the number of LBM grid cells. We used Richardson’s method^30^ to evaluate the convergence error of the LBM method, while the exact solution of the EM energy density is given by:

7 $$\mathrm{U}=\underset{{\delta x}\to 0}{{\lim}}\mathrm{U}\left(\delta x\right)\approx \frac{{2}^{n}U\left(\frac{\delta x}{2}\right)-U\left(\delta x\right)}{{2}^{n}-1}+\mathrm{L}\left({\delta x}^{n+1}\right),$$

with an n + 1 order of error $\mathrm{L}({\delta x}^{n+1})$, where $\mathrm{\delta x}=\frac{L}{N}$, as $L$ is the length of the computational domain, and N is the number of cells. Therefore, the error of the model is at the second-order, and the relative errors can be evaluated as

8 $$\mathrm{L}1=\frac{1}{N}\sum_{i=1}^{N}|\frac{\mathrm{U}\left(\delta x\right)-\mathrm{U}}{\mathrm{U}}|.$$

Figure 5 shows that the relative error decreases with an increase in LBM grid cell number as ${\mathrm{\delta x}}^{1.92}$. The decrease in error, supports that the present scheme has a second-order convergence.

### Effects of film thickness and frequency on Gaussian modulated sinusoidal EM pulse

In light of the validations results presented in sect. “EM wave in Debye medium based on LBM method”, the adaptability of the LBM-SEF model is further assessed by examining two intriguing phenomena of the response of dispersive medium to an incoming EM wave. Here we focus on a scenario where an EM pulse travels through the air and encounters a Debye medium. As depicted in Fig. 6, the simulation domain consists of 10,000 lattice cells, each with a uniform size of 3.75 $\mathrm{\mu m}$. The EM pulse in the air is modeled by a Gaussian modulated sinusoidal pulse, with its amplitude given by:

9 $$\mathrm{E}\left(\mathrm{x},\mathrm{t}\right)={E}_{M}\mathrm{sin}\left(2\mathrm{\pi \omega t}\right)\mathrm{exp}\left(-{\left[\frac{x-{x}_{c}}{\alpha }\right]}^{2}\right).$$

**Figure 7:**
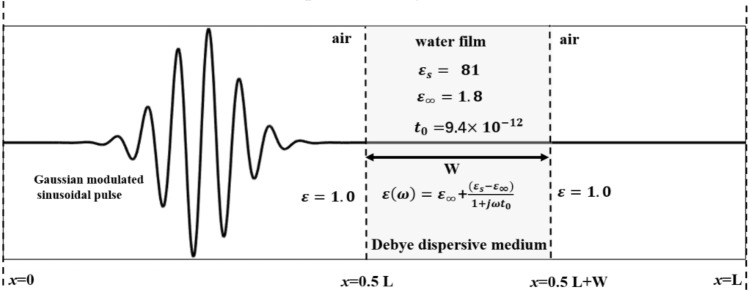
Schematic view of the computational domain of a sinusoidal modulated Gaussian pulse passing through a dispersive film.

Here ${E}_{M}$ is the electric field amplitude of the pulse,$\upomega$ is 0.2 or 0.8 THz for the low- and high-frequency cases, respectively. The pulse incidents from the air (right), pass through the Debye film (middle), and return back to air, as depicted in Fig. 7.

**Figure 8:**
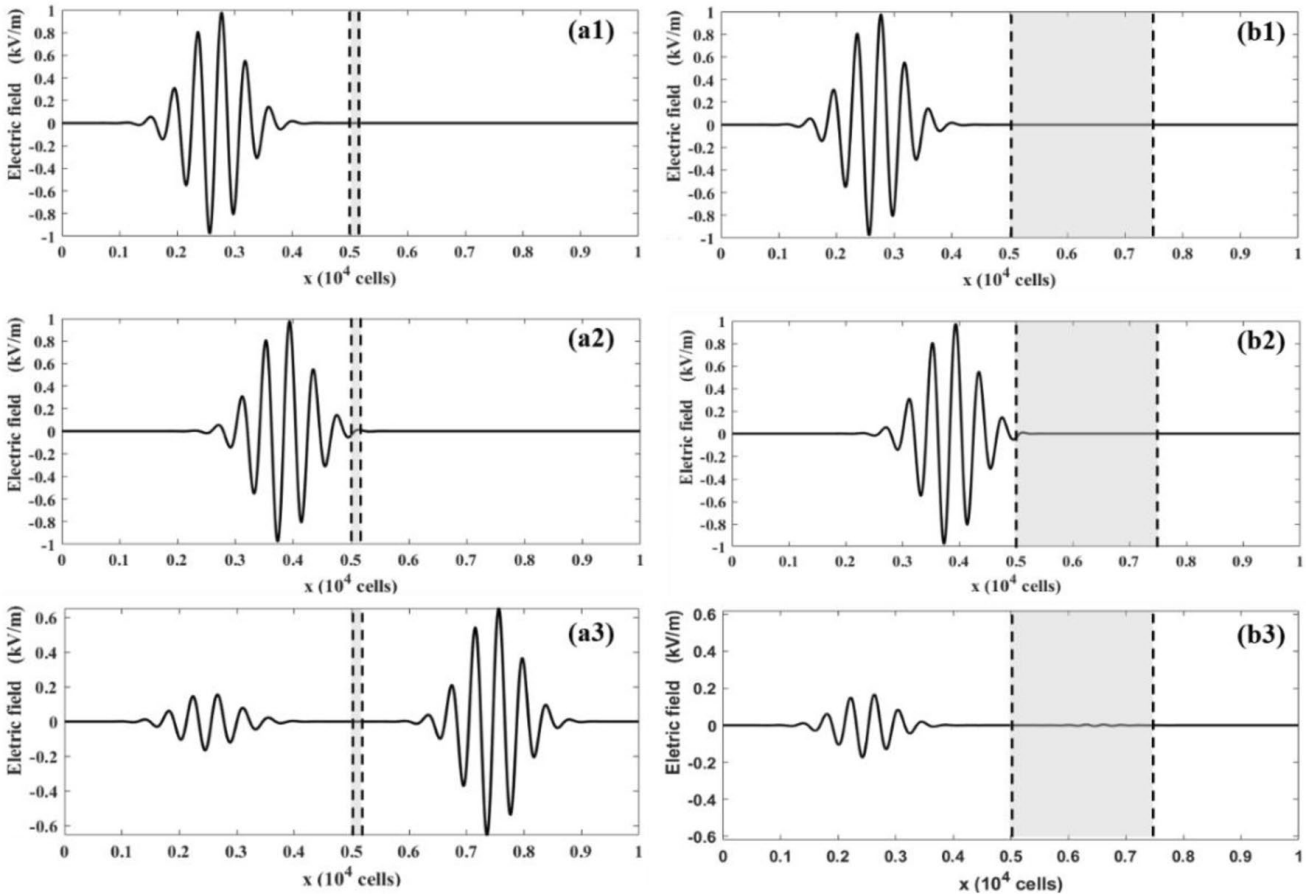
The distribution of the electric fields at different times and film thicknesses: (**a1**) 0 ps, 1000 cells; (**a2**) 33 ps, 1000 cells; (**a3**) 56 ps, 1000 cells; (**b1-b3**) the same to (**a1**-**a3**), but for film thickness of 2000 cells.

Depending on the file thickness, the EM wave can be partially or completely aborted by the Debye medium as it passes through it. The simulation results for thin- and thick- films of Debye media are presented in Fig. 8. Figure 8a1,a2 and b1,b2 show that, at time = 0 ps, the EM wave is in the air domain (Fig. 8a1 and b1). Then, it touches the air–water interface at approximately time = 33 ps (Fig. 8a2 and b2). At time = 56 ps, Fig. 8a3 shows that the EM wave is partially absorbed by the thin water film, while Fig. 8b3 shows that the EM wave is almost completely absorbed by the thick water film.

**Figure 9:**
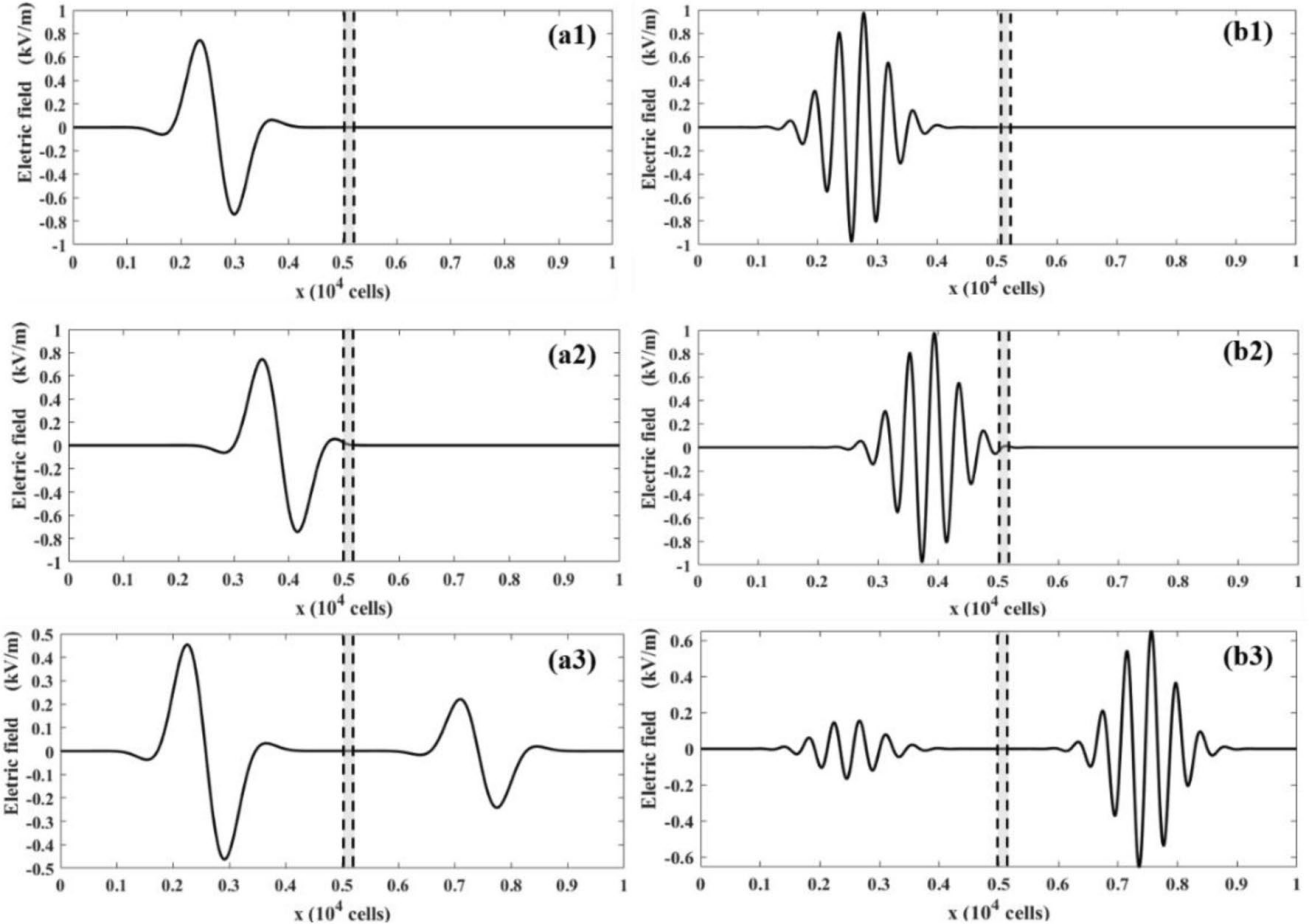
The distribution of electric fields at different times and pulse frequencies: (**a1**) 0 ps, 0.5 THz; (**a2**) 33 ps, 0.5 THz; and (**a3**) 56 ps, 0.5 THz; (**b1-b3**) the same to (**a1**-**a3**), but for pulse frequency of 2.0 THz.

The frequency also significantly affects the EM wave propagation. The distribution of the electric fields of the low- and high-frequency Gaussian modulated sinusoidal pulses are depicted in Fig. 9. Figure 9a1 and b1 show that the EM wave at low frequency is in air domain at time = 0 ps, and it then starts touching the air–water interface at time = 33 ps (Fig. 9a2,b2). At time t = 56 ps, the EM wave is split into two constituents: the left in the air is reflected by the Debye medium and travels to the left, while the transmitted wave travels to the right. A comparison between Fig. 9a3 and b3 indicates that the Debye film is more transparent for higher-frequency EM waves. The decrease of the transparency with the EM frequency is relatively low. These results further validate the LBM-SEF method for EM wave propagation in dispersive media.

## Conclusions

In this manuscript, we proposed and implemented a novel lattice Boltzmann method with an extended force term to simulate the EM wave propagation in dispersive media. The proposed method is mathematically consistent with Maxwell’s equations and the accuracies were demonstrated by comparing simulation results with those of the (FD)^2^TD method and the previous lattice Boltzmann method using pseudo permittivity. Two typical yet intriguing cases were employed to assess the ability and adaptability of the new method for EM waves traveling through Debye films. The results confirmed the accuracy and robustness of the new method for dealing with frequency-dependent reflection and transmission at the air–water interface. Furthermore, the results can aid in constructing two-dimensional and three-dimensional lattice Boltzmann method for the simulation of EM waves in dispersive media.

## Materials and methods

The Lattice Boltzmann method (LBM) is widely used to describe the evolution of particle distributions with discrete space and time coordiantes^19^. The density distribution and the particle velocities can be well reproduced by the density and moments of the model^20,21^. Here we briefly describe the technical details of the extended LBM equation with a force term to describe EM wave propagation in dispersive media. We also demonstrated that the new method is consistent with Maxwell equations by using the Chapman-Enskog expansion technique.

### Governing equations

We start from the time-dependent Maxwell equations in dielectric media at position x and time t^28^:

10 $$\nabla \times \mathbf{E}\left(x,t\right)=-\frac{\partial \mathbf{B}}{\partial t}, \nabla \times \mathbf{H}\left(x,t\right)=-\frac{\partial \mathbf{D}\left({\varvec{x}},{\varvec{t}}\right)}{\partial t}$$

where $\mathbf{H}$ and $\mathbf{E}$ denote the intensities of the magnetic and electric fields, respectively, $\mathbf{B}$ and $\mathbf{D}$ are the magnetic induction and electric displacement,and **J** is the current density. $\mathbf{B}$ and $\mathbf{H}$, $\mathbf{D}$ and $\mathbf{E}$ are related by $\mathbf{B}={\varvec{\upmu}}\mathbf{H}$, and $\mathbf{D}={\varvec{\upvarepsilon}}\mathbf{E}$**,** where ${\varvec{\upmu}}$ and ${\varvec{\upvarepsilon}}$ are the permittivity and permeability of the medium, respectively. Furthermore, ${\varvec{\upmu}}$ and ${\varvec{\upvarepsilon}}$ can also be related to the relative constants ${{\varvec{\upmu}}}_{r}$,${\boldsymbol{ }{\varvec{\varepsilon}}}_{{\varvec{r}}}$ by ${\varvec{\upmu}}$**=**${{\varvec{\upmu}}}_{r}{{\varvec{\upmu}}}_{0}$ and ${\varvec{\upvarepsilon}}$**=**${{\varvec{\varepsilon}}}_{{\varvec{r}}}{{\varvec{\varepsilon}}}_{0}$.

As discussed in the study by Chen et al.^12^, the permittivity of a linear, isotropic dispersive medium, ${\varvec{\upvarepsilon}}({\varvec{\upomega}})$, relates **D** and **E** in the frequency domain via D(${\varvec{\upomega}}$) = ${\varvec{\upvarepsilon}}({\varvec{\upomega}})\mathbf{E}({\varvec{\upomega}})$. While in the time domain, we have

11 $$\mathbf{D}\left(x,t\right)={\upvarepsilon }_{\infty }{\upvarepsilon }_{0}\mathbf{E}\left(x,t\right)+{\upvarepsilon }_{0}{\int }_{0}^{\mathrm{t}}\mathbf{E}(x,t-\tau )\upzeta (\tau )\mathrm{d}\tau .$$

Here ${\upvarepsilon }_{\infty }$ is the permittivity at infinite high frequency, $\upzeta (\tau )$ is the time domain electric susceptibility. Different from the work by Chen et al.^12^**,** in this study, we use the following expression:

$${\upvarepsilon }_{\infty }{\upvarepsilon }_{0}\frac{\partial \mathbf{E}\left({\varvec{x}},{\varvec{t}}\right)}{\partial t} = \nabla \times \mathbf{H}\left(x,t\right)-{\upvarepsilon }_{0}\frac{\partial }{\partial t}{\int }_{0}^{t}\mathbf{E}\left(x,t-\tau \right)\upzeta \left(\uptau \right)\mathrm{d}\tau ,$$

12 $$\frac{\partial \mathbf{B}}{\partial t}=-\nabla \times \mathbf{E}\left(x,t\right).$$

In the previous method by Chen et al.^12^, it is necessary to evaluate the pseudo permittivity for the simulation of the EM wave propagation, which significantly influences the overall accuracy of the simulation via the selection of the time resolution $\Delta \mathrm{t}$^12^. However, according to Eq. (12), the evaluation of the pseudo permittivity is no longer required in our method. As will be demonstrated in the following, only a single force term would be sufficient for the simulation.

In a one dimensional dispersive medium, the model can be further simplified for plane EM wave propagation:

$${\upvarepsilon }_{\infty }{\upvarepsilon }_{0}\frac{\partial {\mathbf{E}}_{{\varvec{y}}}\left({\varvec{x}},{\varvec{t}}\right)}{\partial t} = \frac{\partial {\mathbf{H}}_{\mathbf{z}}({\varvec{x}},{\varvec{t}})}{\partial x}-{\upvarepsilon }_{0}\frac{\partial }{\partial \mathrm{t}}{\int }_{0}^{\mathrm{t}}\mathbf{E}\left(x,t-\uptau \right)\upzeta \left(\uptau \right)\mathrm{d\tau },$$

13 $${\varvec{\upmu}}\frac{\partial {\mathbf{H}}_{\mathbf{z}}\left(x,t\right)}{\partial t}=-\frac{\partial {\mathbf{E}}_{{\varvec{y}}}\left(x,t\right)}{\partial x}.$$

It is noted that with Eq. (4), the simplified LBM model is also mathematically more strict.

### Extended lattice Boltzmann model

In this section, we proposed a dimensionless lattice Boltzmann model with a force term for EM waves in a linear dispersive medium. The proposed lattice Boltzmann model is as follows:

14 $${f}_{i}\left(x+{\mathrm{e}}_{\mathrm{i}}\Delta \mathrm{t},t+\Delta t\right)-{f}_{i}\left(x,t\right)=-\frac{\Delta \mathrm{t}}{\uptau }\left({f}_{i}\left(x,t\right)-{f}_{i}^{eq}\left(x,t\right)\right)-\Delta {\mathrm{tF}}_{\mathrm{i}}\left(x,t\right)\mathrm{ i}=\mathrm{0,1},2,\dots ,\mathrm{ b}$$

where ${f}_{i}\left(x,t\right)$ is the particle distribution function at position *x*, time *t* in the i-th direction. ${\mathrm{e}}_{\mathrm{i}}$ is the lattice vector in the *i*-th direction, $\Delta \mathrm{t}$ is the time step, $\tau$ is the relaxation time, and b indexes the lattice vectors. Obviously, b = 2 for one-dimensional media. ${f}_{i}^{eq}\left(x,t\right)$ is the local distribution at equilibrium, and ${\mathrm{F}}_{\mathrm{i}}\left(x,t\right)$ is a force term.

The macroscopic EM characteristics are given by:

15 $${\upvarepsilon }_{\infty }{\upvarepsilon }_{0}{\mathbf{E}}_{{\varvec{y}}}\left({\varvec{x}},{\varvec{t}}\right)=\sum_{i}{f}_{i}\left(x,t\right) {\mathbf{H}}_{\mathbf{z}}\left(x,t\right)=\sum_{i}{{\mathrm{e}}_{\mathrm{i}}f}_{i}\left(x,t\right)$$

To solve the EM wave equation, we employed the LBM scheme to calculate the bandgap of photonic materials. Furthermore, the ${f}_{i}^{eq}\left(\mathrm{x},\mathrm{t}\right)$ is written as:

16 $${f}_{i}^{eq}\left(x,t\right)={A}_{i}{\upvarepsilon }_{\infty }{\upvarepsilon }_{0}{\mathbf{E}}_{{\varvec{y}}}\left({\varvec{x}},{\varvec{t}}\right)+{\mathrm{B}}_{i}{\mathrm{e}}_{\mathrm{i}}{\mathbf{H}}_{\mathbf{z}}\left(x,t\right)$$

where ${A}_{i}$ and ${B}_{i}$ are weighting parameters. According to symmetrical properties, the distribution function at equilibrium, ${f}_{i}^{eq}\left(\mathrm{x},\mathrm{t}\right)$ can be written as:

$${f}_{i}^{eq}\left(\mathrm{x},\mathrm{t}\right)={A}_{0}{{\upvarepsilon }_{\infty }{\upvarepsilon }_{0}\mathbf{E}}_{{\varvec{y}}}\left({\varvec{x}},{\varvec{t}}\right), i=0$$

17 $${f}_{i}^{eq}\left(x,t\right)=A{{\upvarepsilon }_{\infty }{\upvarepsilon }_{0}\mathbf{E}}_{{\varvec{y}}}\left({\varvec{x}},{\varvec{t}}\right)+B{\mathrm{e}}_{\mathrm{i}}{\mathbf{H}}_{\mathbf{z}}\left(x,t\right), i>0$$

Another two conservation constraints were used to specify the distribution weights:

$$\sum_{i}{f}_{i}^{eq}(x,t)=\sum_{i}{f}_{i}\left(x,t\right)={\upvarepsilon }_{\infty }{\upvarepsilon }_{0}{\mathbf{E}}_{{\varvec{y}}}\left(\mathbf{x},\mathbf{t}\right)$$

18 $$\sum_{i}{\mathrm{e}}_{\mathrm{i}}{f}_{i}^{eq}(x,t)=\sum_{i}{{\mathrm{e}}_{\mathrm{i}}f}_{i}\left(x,t\right)={\mathbf{H}}_{\mathbf{z}}\left(x,t\right)$$

Based on Eqs. (17) and (18), we obtain the following equations:

19 $${A}_{0}=1-2A B=1/2$$

### Chapman-Enskog expansion

The distribution function ${f}_{i}\left(x,t\right)$ can be expanded up to the second-order according to the Chapman-Enskog expansion:

20 $${f}_{i}\left(x,t\right)={f}_{i}^{eq}\left(x,t\right)+\uptheta {f}_{i}^{1}\left(x,t\right)+{\uptheta }^{2}{f}_{i}^{2}\left(x,t\right)+O({\uptheta }^{3})$$

where the expansion parameter $\uptheta$ is usually understood as Knudsen number [40], while it is not necessary to calculate this parameter using the Chapman-Enskog expansion technique.

It shouble be noted that in Eq. (20), ${f}_{i}^{1}\left(x,t\right)$ and ${f}_{i}^{2}\left(x,t\right)$ can be viewed as formal expansion functions corresponding to distribution functions at different scales. Combining Eqs. (18), (19) and (20), we obtain the following equations:

$$\sum_{i}{f}_{i}\left(x,t\right)=\sum_{i}{f}_{i}^{0}\left(x,t\right)+\uptheta {f}_{i}^{1}\left(x,t\right)+{\uptheta }^{2}{f}_{i}^{2}\left(x,t\right)+O({\uptheta }^{3})=\sum_{i}{f}_{i}^{eq}(x,t)$$

21 $$\sum_{i}{{\mathrm{e}}_{\mathrm{i}}f}_{i}\left(x,t\right)=\sum_{i}{\mathrm{e}}_{\mathrm{i}}{f}_{i}^{eq}\left(x,t\right)+\uptheta {\mathrm{e}}_{\mathrm{i}}{f}_{i}^{1}\left(x,t\right)+{\uptheta }^{2}{\mathrm{e}}_{\mathrm{i}}{f}_{i}^{2}\left(x,t\right)+{\mathrm{e}}_{\mathrm{i}}O({\uptheta }^{3})=\sum_{i}{\mathrm{e}}_{\mathrm{i}}{f}_{i}^{eq}(x,t)$$

where ${f}_{i}^{0}\left(x,t\right)={f}_{i}^{eq}\left(x,t\right)$. Given that ${\mathrm{e}}_{0}$=0and $\uptheta$ is an infinitesimal, we arrive at

$$\sum_{i}{f}_{i}^{k}\left(x,t\right)=0$$

22 $$\sum_{i}{\mathrm{e}}_{\mathrm{i}}{f}_{i}^{k}\left(x,t\right)=0,\mathrm{ for }k>0$$

Equation (14) can be Taylor expanded with respect to time,

23 $${f}_{i}\left(x+{\mathrm{e}}_{\mathrm{i}}\Delta t,t+\Delta t\right)-{f}_{i}\left(x,t\right)=\Delta t\left({\partial }_{t}+{\mathrm{e}}_{\mathrm{i\alpha }}{\partial }_{\mathrm{x\alpha }}\right){f}_{i}\left(x,t\right)+\frac{{\Delta t}^{2}}{2}{\left({\partial }_{t}+{\mathrm{e}}_{\mathrm{i\alpha }}{\partial }_{\mathrm{x\alpha }}\right)}^{2}{f}_{i}\left(x,t\right)+o\left({\Delta t}^{3}\right)$$

The force term in Eq. (14) has the following form:

$${\mathrm{F}}_{\mathrm{i}}\left(x,t\right)=\frac{\partial }{\partial t}{\int }_{t=0}^{t}{\mathrm{F}}_{\mathrm{i}}\left(x,\tau \right)d\tau =\frac{\partial }{\partial t}\widetilde{{\mathrm{F}}_{\mathrm{i}}}\left(x,t\right)$$

24 $$\widetilde{{\mathrm{F}}_{\mathrm{i}}}\left(x,t\right)={\int }_{t=0}^{t}{\mathrm{F}}_{\mathrm{i}}\left(x,\tau \right)d\tau$$

Using Chapman-Enskog expansion, we equate the same orders of ${\partial }_{\mathrm{t}}$ and ${\partial }_{\mathrm{x\alpha }}$, which gives :

$${\partial }_{t}=\uptheta {\partial }_{{t}^{(0)}}+{\uptheta }^{2}{\partial }_{{t}^{(1)}}+o({\uptheta }^{3})$$

25 $${\partial }_{\mathrm{x\alpha }}=\uptheta {\partial }_{{\mathrm{x\alpha }}^{(1)}}+o({\uptheta }^{2})$$

Assuming the force term takes the form of:

26 $$\widetilde{{\mathrm{F}}_{\mathrm{i}}}\left(x,\tau \right)=\uptheta \widetilde{{\mathrm{F}}_{{i}^{(1)}}}\left(x,\tau \right)$$

Combining Eqs. (25) and (26), and substitute into Eq. (14), we have:

$$\Delta t\left({\partial }_{t}+{\mathrm{e}}_{\mathrm{i\alpha }}{\partial }_{\mathrm{x\alpha }}\right){f}_{i}\left(x,t\right)+\frac{{\Delta t}^{2}}{2}{\left({\partial }_{t}+{\mathrm{e}}_{\mathrm{i\alpha }}{\partial }_{\mathrm{x\alpha }}\right)}^{2}{f}_{i}\left(x,t\right)+o\left({\Delta t}^{3}\right)$$

 = $$-\frac{\Delta t}{\tau }\left({f}_{i}\left(x,t\right)-{f}_{i}^{0}\left(x,t\right)\right)-\Delta {\mathrm{tF}}_{\mathrm{i}}\left(x,t\right)$$

 = $$-\frac{\Delta t}{\uptau }\left(\left({f}_{i}^{0}\left(x,t\right)+\uptheta {f}_{i}^{1}\left(x,t\right)+{\uptheta }^{2}{f}_{i}^{2}\left(x,t\right)+O\left({\uptheta }^{3}\right)\right)-{f}_{i}^{0}\left(x,t\right)\right)-\Delta t\uptheta {\mathrm{F}}_{{\mathrm{i}}^{(1)}}\left(x,t\right)$$

27 $$=-\frac{\Delta t}{\uptau }\left(\uptheta {f}_{i}^{1}\left(x,t\right)+{\uptheta }^{2}{f}_{i}^{2}\left(x,t\right)+O\left({\uptheta }^{3}\right)\right)-\Delta t(\uptheta {\partial }_{{\mathrm{t}}^{\left(0\right)}}+{\uptheta }^{2}{\partial }_{{\mathrm{t}}^{\left(1\right)}}+o({\uptheta }^{3}))(\uptheta \widetilde{{\mathrm{F}}_{{i}^{(1)}}}\left(x,t\right))$$

Grouping terms with same orders of $\uptheta$ and ${\uptheta }^{2}$:

$${\partial }_{{\mathrm{t}}^{(0)}}{f}_{i}^{0}\left(x,t\right)+{\mathrm{e}}_{\mathrm{i\alpha }}{\partial }_{{\mathrm{x\alpha }}^{(1)}}{f}_{i}^{0}\left(x,t\right)=-\frac{1}{\uptau }{f}_{i}^{1}\left(x,t\right)$$

28 $${\partial }_{{t}^{(1)}}{f}_{i}^{0}\left(x,t\right)+\left(-\uptau +\frac{\Delta t}{2}\right){\left({\partial }_{{t}^{\left(0\right)}}+{\mathrm{e}}_{\mathrm{i\alpha }}{\partial }_{{\mathrm{x\alpha }}^{\left(1\right)}}\right)}^{2}{f}_{i}^{0}\left(x,t\right)=-\frac{1}{\uptau }{f}_{i}^{2}\left(x,t\right)-{\partial }_{{\mathrm{t}}^{\left(0\right)}}\widetilde{{\mathrm{F}}_{{i}^{(1)}}}\left(\mathrm{x},t\right)$$

Summing Eq. (28) over *i*, and using Eqs. (15) and (22), we could get that:

29 $${\partial }_{{\mathrm{t}}^{(0)}}\sum_{i}{f}_{i}^{0}\left(x,t\right)+{\partial }_{{\mathrm{x\alpha }}^{(1)}}\sum_{i}{\mathrm{e}}_{\mathrm{i\alpha }}{f}_{i}^{0}\left(x,t\right)=-\frac{1}{\uptau }\sum_{i}{f}_{i}^{1}\left(x,t\right)={\partial }_{{\mathrm{t}}^{(0)}}{\upvarepsilon }_{\infty }{\upvarepsilon }_{0}{\mathbf{E}}_{{\varvec{y}}}\left(x,t\right)+{\partial }_{{\mathrm{x\alpha }}^{(1)}}{\mathbf{H}}_{\mathbf{z}}\left(x,t\right)=0$$

and

30 $${\partial }_{{t}^{\left(1\right)}}{\upvarepsilon }_{\infty }{\upvarepsilon }_{0}{\mathbf{E}}_{{\varvec{y}}}\left(x,t\right)+\left(-\uptau +\frac{\Delta t}{2}\right)\left(\begin{array}{l}{\partial }_{{\mathrm{t}}^{\left(0\right)}}{\partial }_{{\mathrm{t}}^{\left(0\right)}}{\upvarepsilon }_{\infty }{\upvarepsilon }_{0}{\mathbf{E}}_{{\varvec{y}}}\left(x,t\right)\\ +2{{\partial }_{{t}^{\left(0\right)}}\partial }_{{\mathrm{x\alpha }}^{\left(1\right)}}{\mathbf{H}}_{\mathbf{z}}\left(x,t\right)+{\partial }_{{\mathrm{x\alpha }}^{\left(1\right)}}{\partial }_{{\mathrm{x\alpha }}^{\left(1\right)}}2A{\upvarepsilon }_{\infty }{\upvarepsilon }_{0}{\mathbf{E}}_{{\varvec{y}}}\left(x,t\right)\end{array}\right)=-{\partial }_{{t}^{\left(0\right)}}{\sum }_{i}\widetilde{{\mathrm{F}}_{{i}^{(1)}}}\left(x,t\right)$$

By multipling Eqs. (29) and (30) by ${\mathrm{e}}_{\mathrm{i\alpha }}$ and considering (22) we obtain:

$${\partial }_{{t}^{(0)}}\sum_{i}{\mathrm{e}}_{\mathrm{i\alpha }}{f}_{i}^{0}\left(x,t\right)+{\partial }_{{\mathrm{x\alpha }}^{(1)}}\sum_{i}{{\mathrm{e}}_{\mathrm{i\alpha }}\mathrm{e}}_{\mathrm{i\alpha }}{f}_{i}^{0}\left(x,t\right)=-\frac{1}{\uptau }\sum_{i}{\mathrm{e}}_{\mathrm{i\alpha }}{f}_{i}^{1}\left(x,t\right)$$

31 $${\partial }_{{t}^{(0)}}{\mathbf{H}}_{\mathbf{z}}\left(x,t\right)+{\partial }_{{\mathrm{x\alpha }}^{(1)}}2\mathrm{A}{\upvarepsilon }_{\infty }{\upvarepsilon }_{0}{\mathbf{E}}_{{\varvec{y}}}\left(x,t\right)=0$$

32 $${\partial }_{{t}^{\left(1\right)}}{\mathbf{H}}_{\mathbf{z}}\left(x,t\right)+\left(-\uptau +\frac{\Delta t}{2}\right)\left({\mathbf{H}}_{\mathbf{z}}\left(x,t\right)+{{4\partial }_{{t}^{\left(0\right)}}\partial }_{{\mathrm{x\alpha }}^{\left(1\right)}}{\upvarepsilon }_{\infty }{\upvarepsilon }_{0}{\mathbf{E}}_{{\varvec{y}}}\left(x,t\right)+{\partial }_{{\mathrm{x\alpha }}^{\left(1\right)}}{\partial }_{{\mathrm{x\alpha }}^{\left(1\right)}}{\mathbf{H}}_{\mathbf{z}}\left(x,t\right)\right)=-{\partial }_{{t}^{\left(0\right)}}{\sum }_{i}{\mathrm{e}}_{\mathrm{i\alpha }}\widetilde{{\mathrm{F}}_{{i}^{(1)}}}\left(x,t\right)$$

Comparing the above equation with the original Maxwell Eq. (4), we have the constraints:

$${\sum }_{i}\widetilde{{\mathrm{F}}_{{i}^{(1)}}}\left(x,t\right)={\upvarepsilon }_{0}{\int }_{0}^{t}\mathbf{E}\left(x,t-\uptau \right)\upzeta \left(\uptau \right)\mathrm{d\tau },$$

$$2\mathrm{A}{\upvarepsilon }_{\infty }{\upvarepsilon }_{0}={\varvec{\upmu}},$$

$$\uptau =\frac{\Delta t}{2},$$

33 $${\sum }_{i}{\mathrm{e}}_{\mathrm{i\alpha }}\widetilde{{\mathrm{F}}_{{i}^{(1)}}}\left(x,\mathrm{t}\right)=0.$$

By considering (θ × Eq. (29) + θ^2^ × Eq. (30)), we obtain:

$${\upvarepsilon }_{\infty }{\upvarepsilon }_{0}{\partial }_{\mathrm{t}}{\mathbf{E}}_{{\varvec{y}}}\left(x,t\right)=-{{\partial }_{\mathrm{x}}\mathbf{H}}_{\mathbf{z}}\left(x,t\right)-{\upvarepsilon }_{0}{\partial }_{\mathrm{t}}{\int }_{0}^{t}\mathbf{E}\left(x,t-\uptau \right)\upzeta \left(\uptau \right)\mathrm{d\tau }+O\left({\Delta t}^{3}\right)+O\left({\uptheta }^{3}\right),$$

34 $${{\varvec{\upmu}}\partial }_{\mathrm{t}}{\mathbf{H}}_{\mathbf{z}}\left(x,t\right)=-{\partial }_{\mathrm{x}}{\mathbf{E}}_{{\varvec{y}}}\left(x,t\right)+O\left({\Delta t}^{3}\right)+O\left({\uptheta }^{3}\right).$$

Based on Eq. (34), it is evident that the proposed LBM scheme with the force term is capable of recovering the Maxwell equations at the limits of $\Delta t$ and $\uptheta$ approach to zero.

Furthermore, the force term $\widetilde{{\mathrm{F}}_{{i}^{(1)}}}\left(\mathrm{x},t\right)$ can be evaluated by the integration

$$\widetilde{{\mathrm{F}}_{1}}\left(x,t\right)=\widetilde{{\mathrm{F}}_{2}}\left(x,t\right)=0,$$

35 $$\widetilde{{\mathrm{F}}_{0}}\left(x,t\right)={\upvarepsilon }_{0}{\int }_{0}^{t}\mathbf{E}\left(x,t-\uptau \right)\upzeta \left(\uptau \right)\mathrm{d\tau }.$$

Based on Eq. (35), we can calculate the forcing term as follows:

$${\mathrm{F}}_{1}\left(x,t\right)={\mathrm{F}}_{2}\left(x,t\right)=0$$

$${\mathrm{F}}_{0}\left(x,t\right)={{\upvarepsilon }_{0}\partial }_{t}{\int }_{0}^{t}\mathbf{E}\left(x,t-\uptau \right)\upzeta \left(\uptau \right)\mathrm{d\tau }={\upvarepsilon }_{0}\mathbf{E}\left(x,0\right)\upzeta \left(t\right)+{\upvarepsilon }_{0}{\int }_{0}^{t}{\partial }_{t}\mathbf{E}(x,t-\uptau )\upzeta (\uptau )\mathrm{d\tau }$$

$$={\upvarepsilon }_{0}\mathbf{E}\left(x,0\right)\upzeta \left(t\right)+{\upvarepsilon }_{0}P\left(x,t\right)$$

36 $${\mathrm{F}}_{0}\left(x,t-\Delta t\right)={\upvarepsilon }_{0}\mathbf{E}\left(x,0\right)\upzeta \left(t\right)+{\upvarepsilon }_{0}P\left(x,t-\Delta t\right),$$

where the force kernel function $P\left(x,t\right)={\int }_{0}^{t}{\partial }_{\mathrm{t}}\mathbf{E}(x,t-\uptau )\upzeta (\uptau )\mathrm{d\tau }$, which will be calculated in sect. “Calculation of the force term of Debye media”.

### Calculation of the force term of Debye media

The Debye model in the time domain (Chen et al.^12^) is given by:

37 $$\upzeta \left(\mathrm{t}\right)=\frac{{\varepsilon }_{s}-{\varepsilon }_{\infty }}{{t}_{0}}{e}^{-\frac{t}{{t}_{0}}}S\left(t\right),$$

where ${\varepsilon }_{s}$ denotes the static permittivity, ${\varepsilon }_{\infty }$ denotes the infinite frequency permittivity, ${t}_{0}$ denotes the relaxation time constant, and $S\left(t\right)$ denotes the unit step function.

Based on Eqs. (27) and (28), the forcing term is determined by $P\left(x,t\right)$ as follows:

$$P\left(x,t\right)={\int }_{0}^{t}{\partial }_{t}\mathbf{E}(x,t-\uptau )\upzeta (\uptau )\mathrm{d\tau }={\int }_{0}^{\mathrm{n}\Delta t}{\partial }_{t}\mathbf{E}(x,\mathrm{n}\Delta t-\uptau )\upzeta (\uptau )\mathrm{d\tau }$$

$$=\sum_{m=0}^{n}{\partial }_{t}\mathbf{E}(x,(\mathrm{n}-\mathrm{m})\Delta t){\int }_{m\Delta t}^{(m+1)\Delta t}\upzeta (\uptau )\mathrm{d\tau }$$

$$=\sum_{m=0}^{n}{\partial }_{t}\mathbf{E}(x,(\mathrm{n}-\mathrm{m})\Delta t){(\varepsilon }_{s}-{\varepsilon }_{\infty }){e}^{-\frac{t}{{t}_{0}}}{|}_{m\Delta t}^{(m+1)\Delta t}$$

$$=\sum_{m=0}^{n}{\partial }_{t}\mathbf{E}(x,(\mathrm{n}-\mathrm{m})\Delta t){(\varepsilon }_{s}-{\varepsilon }_{\infty }){e}^{-\frac{m\Delta t}{{t}_{0}}}(1-{e}^{-\frac{\Delta t}{{t}_{0}}})$$

$$=\sum_{q=0}^{n}{\partial }_{t}\mathbf{E}(x,\mathrm{q}\Delta t){(\varepsilon }_{s}-{\varepsilon }_{\infty }){e}^{-\frac{(n-q)\Delta t}{{t}_{0}}}(1-{e}^{-\frac{\Delta t}{{t}_{0}}})$$

38 $$=\sum_{q=0}^{n}\frac{\mathbf{E}\left(x,\mathrm{q}\Delta t\right)-\mathbf{E}\left(x,\mathrm{q}\Delta t-\Delta t\right)}{\Delta \mathrm{t}}{(\varepsilon }_{s}-{\varepsilon }_{\infty }){e}^{-\frac{(n-q)\Delta t}{{t}_{0}}}(1-{e}^{-\frac{\Delta t}{{t}_{0}}}).$$

Furthermore, the term $P\left(x,t\right)$ can be calculated based on the following.

$$P\left(x,t-\Delta t\right)={\int }_{0}^{t-\Delta t}{\partial }_{t}\mathbf{E}\left(x,t-\uptau \right)\upzeta \left(\uptau \right)\mathrm{d\tau }={\int }_{0}^{\left(\mathrm{n}-1\right)\Delta t}{\partial }_{t}\mathbf{E}\left(x,\mathrm{n}\Delta t-\uptau \right)\upzeta \left(\uptau \right)\mathrm{d\tau }$$

39 $$=\sum_{q=0}^{n-1}\frac{\mathbf{E}\left(x,\mathrm{q}\Delta t\right)-\mathbf{E}\left(x,\mathrm{q}\Delta t-\Delta t\right)}{\Delta \mathrm{t}}{(\varepsilon }_{s}-{\varepsilon }_{\infty }){e}^{-\frac{(n-q)\Delta t}{{t}_{0}}}(1-{e}^{-\frac{\Delta t}{{t}_{0}}})$$

$$P\left(x,t\right)= \sum_{q=0}^{n}\frac{\mathbf{E}\left(x,\mathrm{q}\Delta t\right)-\mathbf{E}\left(x,\mathrm{q}\Delta t-\Delta t\right)}{\Delta t}{(\varepsilon }_{s}-{\varepsilon }_{\infty }){e}^{-\frac{\left(N-q\right)\Delta t}{{t}_{0}}}{|}_{N=n}(1-{e}^{-\frac{\Delta t}{{t}_{0}}})$$

$$-\sum_{q=0}^{n-1}\frac{\mathbf{E}\left(x,\mathrm{q}\Delta t\right)-\mathbf{E}\left(x,\mathrm{q}\Delta t-\Delta t\right)}{\Delta \mathrm{t}}{(\varepsilon }_{s}-{\varepsilon }_{\infty }){e}^{-\frac{\left(M-q\right)\Delta t}{{t}_{0}}}{|}_{M=n-1}(1-{e}^{-\frac{\Delta t}{{t}_{0}}})$$

40 $$=P\left(x,t-\Delta t\right){e}^{-\frac{\Delta t}{{t}_{0}}}+\frac{\mathbf{E}\left(x,\mathrm{q}\Delta t\right)-\mathbf{E}\left(x,\mathrm{q}\Delta t-\Delta t\right)}{\Delta \mathrm{t}}{(\varepsilon }_{s}-{\varepsilon }_{\infty })(1-{e}^{-\frac{\Delta t}{{t}_{0}}}).$$

## Data Availability

The datasets used and/or analysed during the current study available from the corresponding author on reasonable request.

## List of symbols

$$\mathbf{B}$$: Magnetic induction
$$\mathbf{D}$$: Electric displacement
$\mathbf{E}$: Electric field intensity
$${\mathrm{e}}_{\mathrm{i}}$$: Lattice vector
${E}_{M}$: Magnitude of electric field intensity
${E}_{M}{\prime}$: Magnitude of transmitted electric field intensity
${E}_{M}^{{\prime}{\prime}}$: Magnitude of reflected electric field intensity
${\mathrm{F}}_{\mathrm{i}}$: Dispersive forcing term
$\widetilde{{\mathrm{F}}_{{i}^{(1)}}}$: Dispersive forcing term
${f}_{i}$: Particle distribution function
$\mathbf{H}$: Magnetic field intensity
${H}_{M}$: Magnetic field intensity
$$k$$: Wave number
$K$: Normalized wave number
$L$: Length of the computational domain
N: Number of cells
$P$: Force kernel function
$S$: Unit step function
${t}_{0}$: Relaxation time constant
$$\Delta \mathrm{t}$$: Time step
$$t$$: Time
U: Electromagnetic energy density
$$W$$: Normalized frequency
$$x$$: Coordinate
${x}_{c}$: Coordinate of pulse center

## Greek symbols

$$\alpha$$: Pulse width
$$\upmu$$: Permittivity
$$\upvarepsilon$$: Permeability
${\varepsilon }_{r}$: Relative permittivity constant
$${\varepsilon }_{s}$$: Static permittivity
${\varepsilon }_{\infty }$: Infinite frequency permittivity
$$\uptheta$$: Expansion parameter
$$\upomega$$: Frequency
$\upzeta$: Electric susceptibility in the time domain
$$\tau$$: Time
